# A Systematic Review and Expert Evaluation of Perioperative SGLT2 Inhibitor‐Associated Ketoacidosis Case Reports

**DOI:** 10.1111/aas.70254

**Published:** 2026-05-13

**Authors:** L. I. P. Snel, X. Li, F. Jamaludin, S. E. Siegelaar, F. Holleman, T. M. Vriesendorp, J. H. DeVries, J. B. L. Hoekstra, B. Preckel, D. H. van Raalte, J. Hermanides, A. H. Hulst

**Affiliations:** ^1^ Department of Anesthesiology Amsterdam UMC, Location AMC, University of Amsterdam Amsterdam the Netherlands; ^2^ Department of Internal Medicine Amsterdam UMC, Location AMC, University of Amsterdam Amsterdam the Netherlands; ^3^ Amsterdam Public Health Research Institute, Amsterdam UMC Amsterdam the Netherlands; ^4^ Amsterdam Cardiovascular Sciences Research Institute, VU University Amsterdam the Netherlands; ^5^ Center for Cell Lineage and Development, Guangzhou Institutes of Biomedicine and Health, Chinese Academy of Sciences Guangzhou China; ^6^ Amsterdam Medical Library, Amsterdam UMC Amsterdam the Netherlands; ^7^ Department of Endocrinology and Metabolism Amsterdam UMC, Location AMC, University of Amsterdam Amsterdam the Netherlands; ^8^ Amsterdam Gastroenterology, Endocrinology, and Metabolism, Amsterdam UMC Amsterdam the Netherlands; ^9^ Department of Internal Medicine Isala Hospital Zwolle the Netherlands

## Abstract

**Introduction:**

The use of sodium–glucose cotransporter‐2 inhibitors (SGLT2i) in the perioperative setting may lead to SGLT2i‐associated postoperative ketoacidosis (SAPKA) in patients with type 2 diabetes (T2D). Therefore, cessation of this drug is recommended before surgery. We aimed to study reported cases to assess the causality of SGLT2i, identify common characteristics, potential risk factors, treatment and outcomes of SAPKA.

**Methods:**

We conducted a systematic literature search to identify case reports of patients with metabolic acidosis and the presence of ketones who used SGLT2i in the perioperative setting. Case reports were summarised for common characteristics, assessed for quality and distributed to a panel of diabetes experts, who evaluated the likelihood of SAPKA using a questionnaire.

**Results:**

Ninety‐three papers containing 128 case reports fulfilled the inclusion criteria. The expert panel found SAPKA to be ‘likely’ in 53 (41%), ‘possible’ in 38 (30%) and ‘unlikely’ in 27 (21%) cases; 10 cases (8%) could not be validated due to insufficient data or implausible timing. SAPKA was therefore considered likely or possible in 71% (91/128) of cases. Common factors identified in the SAPKA reports included a diagnosis of T2D mellitus (*n* = 115), impaired perioperative intake (*n* = 30) and insufficient insulin supplementation (*n* = 10). Treatment with insulin was effective, and ketoacidosis resolved in all surviving patients, although significant morbidity, including ICU admission, was reported in a substantial proportion of cases.

**Discussion:**

Confirming a SAPKA diagnosis is challenging due to the variable reporting quality and numerous confounding factors present during the perioperative period. Clinicians should remain aware of SAPKA given the increasing prevalence of SGLT2i use. Focusing on early recognition and treatment represents a potential alternative strategy to routine preoperative SGLT2i discontinuation, though this requires further prospective evaluation.

**Editorial Comment:**

This systematic review presents an overview and discussion of the many, to date, case reports of ketoacidosis thought to be associated with perioperative SGLT2 inhibitor treatment.

## 1 Introduction

The use of sodium–glucose cotransporter‐2 inhibitors (SGLT2i) has increased in recent years, both as glucose‐lowering agents and for the treatment of heart failure and chronic kidney disease [1, 2, 3]. SGLT2i increase ketogenesis. This results from SGLT2i‐induced glucosuria, which lowers blood glucose, reduces endogenous insulin secretion and increases glucagon, thereby promoting lipolysis and hepatic ketogenesis. In the setting of perioperative fasting and surgical stress, this predisposes treated patients to ketoacidosis even at near‐normal blood glucose concentrations. This is termed SGLT2i‐associated postoperative ketoacidosis (SAPKA). As insulin regulation is central to this phenomenon, this is considered rare in patients without type 2 diabetes mellitus (T2D) [4, 5].

Following case reports of ketoacidosis associated with SGLT2i use, the US Food and Drug Administration (FDA) and the UK Royal College of Anaesthetists recommend a 72‐h cessation of SGLT2i before major surgery [6, 7]. These recommendations are based on the FDA Adverse Event Reporting System, case reports and data from long‐term treatment outcome trials outside the perioperative setting [8, 9]. Although subsequent systematic reviews of SAPKA case reports have described ICU admission rates of 40%–60% in affected patients [10, 11].

However, in the perioperative setting, the risk factors for SAPKA, such as surgical trauma, reduced caloric intake and concurrent infection [8, 12], also cause fasting ketosis, lactic acidosis and renal acidosis. Therefore, the differentiation of perioperative metabolic acidosis challenges the identification of true SAPKA [13]. Furthermore, attributing perioperative ketonemia to SAPKA is challenged by previously observed increases in ketonemia in patients without SGLT2i treatment during cardiac surgery [14].

We were interested in the quality of evidence for the causal link between SGLT2i and perioperative ketoacidosis, common characteristics and the clinical course of cases reported as SAPKA. Therefore, we systematically reviewed the literature, retrieved available case reports related to perioperative ketoacidosis with concurrent SGLT2i use and evaluated common characteristics, precautions, treatments and outcomes. In addition, six diabetes experts evaluated these cases to validate the causal role of SGLT2i in the development of ketoacidosis and the adjudication as SAPKA.

## 2 Methodology

This manuscript was written in accordance with the Preferred Reporting Items for Systematic Reviews and Meta‐Analyses (PRISMA) 2020 statement [15].

### 2.1 Search Strategy and Study Selection

We searched Embase, MEDLINE, CINAHL and the Cochrane Library according to our protocol (published under ID CRD42023384621 at PROSPERO). Our search strategy was performed in line with ‘PICO’ criteria, with terms relating to ‘surgery’ to identify a surgical population, terms relating to ‘SGLT2 inhibitors’ as the intervention and terms relevant to ‘ketoacidosis’ for the outcome (Supporting Information S1). Articles were included from the introduction of SGLT2i in 2013 until the search was performed.

Two independent researchers (L.I.P.S., X.L.) screened all articles in two rounds. The first screening round was based on title and abstract. Only articles that included case reports describing the use of SGLT2i in a perioperative setting were included for further analysis. The second round involved a full‐text analysis, and cases were included when they provided original data and reported the use of SGLT2i in the perioperative setting, the presence of ketones (in urine or blood samples) and acidosis (pH ≤ 7.30) (Supporting Information S3). All duplicates and cases that did not meet these criteria were excluded. Inclusion criteria were deliberately broad, capturing all cases published as perioperative SAPKA regardless of reporting completeness or the plausibility of the reported temporal relationship between surgery and ketoacidosis onset. Cases that could not be validated by the expert panel—due to insufficient biochemical data or implausible timing—were retained and classified accordingly, as the discrepancy between author‐reported attribution and expert‐adjudicated likelihood is itself a central finding of this review. Cases with conflicting assessments that could not be resolved between the first two reviewers were decided upon by a third reviewer (A.H.H.).

### 2.2 Data Extraction

Data extracted included year of publication, first author, baseline demographics (sex, age, body mass index [BMI]), diabetes characteristics (diabetes type, glycated haemoglobin [HbA1c] level, other glucose lowering drugs), type and dosage of SGLT2i, the duration of SGLT2i use, time of last dose of the SGLT2i, time when the SGLT2i were restarted, perioperative insulin use, type of surgery, clinical symptoms, laboratory findings at the time of diagnosis (arterial blood gas analysis, kidney function tests, ketone concentration in urine or serum), treatments given and outcomes reported (mortality and time to resolution of SAPKA). Resolution of SAPKA was defined as the time reported by the case report authors at which metabolic derangements—including pH, bicarbonate and ketone levels—had returned to normal following treatment.

### 2.3 Assessment of the Quality of Reports

Two researchers (L.I.P.S. and X.L.) independently assessed the reporting quality, and a third researcher (A.H.H.) resolved any disagreements. Based on the critical appraisal checklist for case reports by the Joanna Briggs Institute, the case reports were categorised into one of three groups: good, moderate or poor quality of reporting [16]. Higher evaluations were given to accurate descriptions and details about the case, including patient history, current medication, laboratory results, symptoms, treatment and reported outcomes.

### 2.4 Expert Panel Assessment

The full‐text case reports were presented to a panel of six medical consultants, all experts in diabetes (S.E.S., F.H., T.M.V., J.H.D., J.B.L.H., D.H.v.R.). Each case report was randomly presented to two experts, and in the event of disagreement, a third expert was randomly selected to resolve the issue. Each expert was asked to answer the five questions in Table 1 for each assigned case report. (Dropdown menu options can be found in Supporting Information S2.) When a reviewer's answer resulted in a ‘no’ for Question 1 or Question 3, they could select options to support their reasoning using the dropdown menu. Question 2 allowed the selection of one option out of six, while Question 4 allowed a maximum of three options. In addition, based on the questionnaire filled in by the expert panel groups, cases were categorised as ‘likely SAPKA’, ‘possibly SAPKA’, ‘unlikely SAPKA’ and ‘not a ketoacidosis associated with the use of SGLT2i’.

**TABLE 1:** Overview of the questions presented to the expert panel.

Que. stion no.	Question	Type of answers
Q1	Q1: Does this case involve an SGLT‐2 inhibitor‐associated ketoacidosis?	Yes/no
Q2	If you answered ‘No’ to Q1 what is the reason? (If the answer to Q1 was ‘Yes’ skip this question)	Dropdown menu (6 options)^a^
Q3	Is the SGLT‐2i‐associated ketoacidosis related to the operation?	Yes/No
Q4	If you answered: ‘No’ to Q3, what alternative option(s) would you say are more explanatory? (Max. 3 options)	Dropdown menu (22 options)^a^
Q5	Space for additional comments	A free text area for comments

*Note:* Expert panel assessment of all case reports.

^a^ Options can be found in Supporting Information S2.

We systematically studied case reports that described potential SAPKA to evaluate these cases based on the following main questions:

- Could ketoacidosis be confirmed?
- Were SGLT2i the likely cause of ketoacidosis?
- Was surgery the precipitating event?
- Were other factors present that could lead to ketoacidosis?
- What was the reported treatment?

### 2.5 Definition of SAPKA

The definition of SAPKA was based on the criteria for diabetic ketoacidosis (DKA). It is important to distinguish between three levels of case classification used in this review: (i) cases meeting the broad inclusion criteria (acidosis pH < 7.30, presence of ketones and recent SGLT2i use); (ii) the subset fulfilling more stringent objective biochemical criteria confirming a high anion gap metabolic ketoacidosis (pH < 7.3 and anion gap > 12 mEq/L, with plasma ketones > 3.0 mmol/L or elevated urinary ketones) and (iii) the final expert‐panel likelihood classification (likely/possible/unlikely/not SGLT2i‐associated). The inclusion threshold was intentionally broad to capture all cases self‐reported as SAPKA in the literature for expert review. Fulfilling these criteria was considered necessary but not sufficient for a ‘likely SAPKA’ classification: cases could still be rated ‘unlikely SAPKA’ when the clinical context rendered a surgery‐ and SGLT2i‐triggered aetiology implausible [10, 17].

### 2.6 Statistical Analysis

Baseline characteristics of the case reports are presented as means and standard deviations for numerical data or numbers and percentages for categorical data. Data are shown with the number of times this variable was present out of the total number of included case reports. To determine the level of primary inter‐rater agreement among the quality assessor and the expert panel, Fleiss' kappa analyses were performed on the outcomes of the first two raters for the cases. All statistical analyses were performed with IBM SPSS Statistics version 26, New York, USA.

## 3 Results

### 3.1 Case Inclusion

**FIGURE 1:**
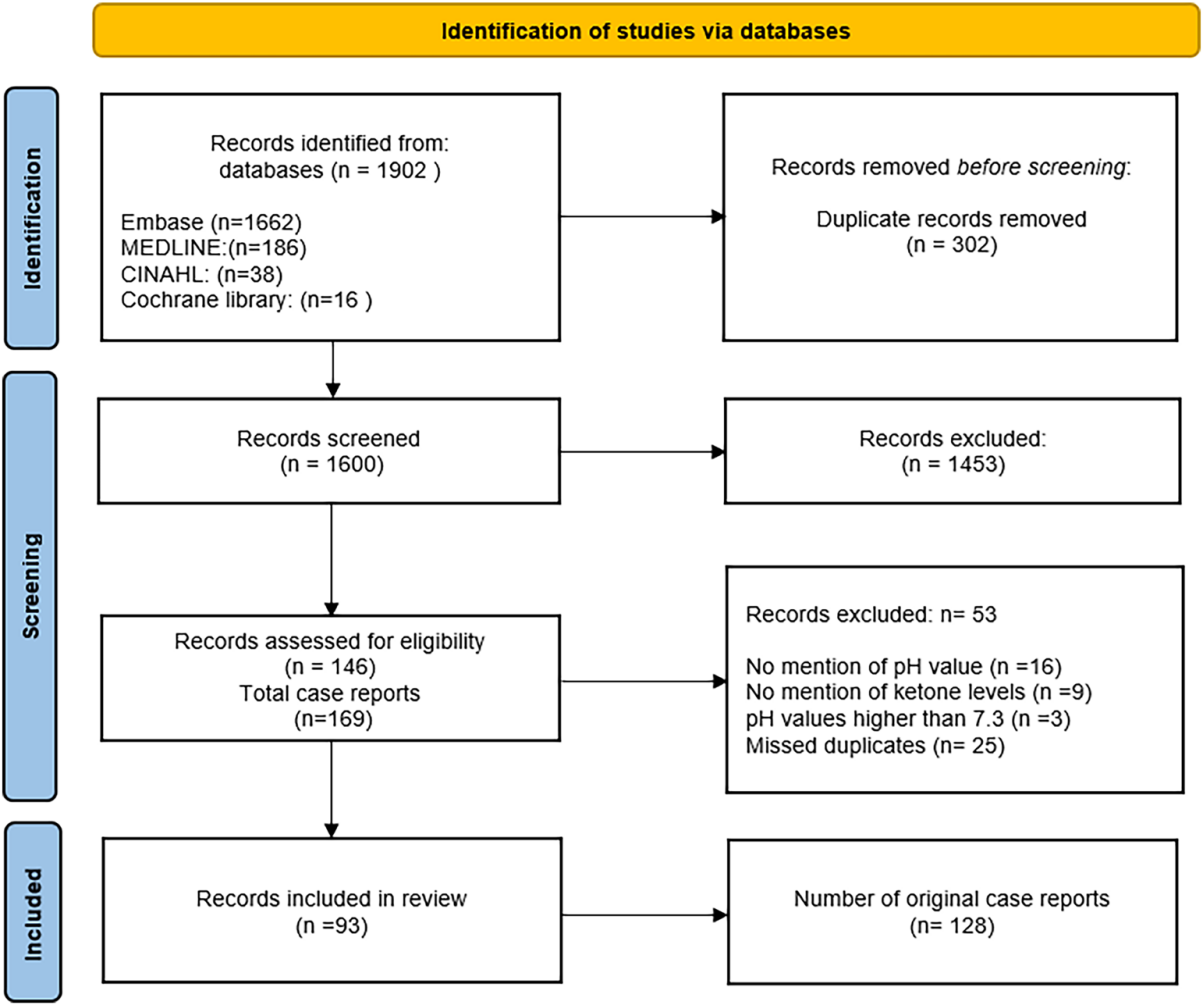
Study inclusion flow chart (PRISMA).

Our search strategy performed on 18 June 2024 yielded 1902 hits (Embase [*n* = 1662], MEDLINE [*n* = 186], CINAHL [*n* = 38], Cochrane Library [*n* = 16]), see Figure 1 and Supporting Information S3. After deduplication, 1612 articles remained, of which 153 article abstracts were deemed eligible and screened in full‐text format. With some papers containing multiple cases, a total of 169 unique case reports were identified. Of these, 41 did not meet our criteria for ketoacidosis and were excluded (Figure 1). The remaining 128 cases were presented to the expert panel. The first case reports were published in 2016, with the majority of yearly cases reported in 2021 (*n* = 32), 2022 (*n* = 20) and 2019 (*n* = 19). Since then, the number of annual cases has decreased, with five cases in 2023 and two cases in 2024.

### 3.2 Quality of Case Reports

The 128 cases presented in our quality assessment were categorised; 43 (33.6%) were deemed ‘good’, 44 (34.4%) were classified as ‘moderate’, and 41 (32.0%) were classified as ‘poor’ quality. We observed a ‘very high level’ of agreement between the two quality assessors (*k* = 0.81, 95% CI = 0.69–0.94, *p* < 0.001).

### 3.3 Case Characteristics

The characteristics of the 128 cases are shown in Table 2. Patients were 56 ± 11 years old, and all were diagnosed with diabetes (99% T2D), with an average HbA1c of 8.5% ± 1.4% (69 ± 16 mmol/mol). Sixteen percent had a known BMI greater than 25 kg/m^2^, with an average BMI of 35.2 ± 8.3 kg/m^2^. Ninety‐five patients (74%) used at least one glucose‐lowering drug other than SGLT2i, of which 33 of 51 cases with available data (65%) used daily insulin injections (26% of the total cohort; mean daily dose 60 ± 49 IU). The most common types of surgeries included bariatric (*n* = 27, 21%), cardiac (*n* = 23, 18%) and abdominal surgery (*n* = 17, 13%).

**TABLE 2:** Patient characteristics of patients with SAPKA.

Patient characteristics	Result	Data available—no. (%)
Sex (female)—no. (%)	64 (54)	118 (92)
Age (years)—*m* ± SD	56.4 ± 11.0	119 (93)
BMI (kg/m^2^)—*m* ± SD	35.2 ± 8.3	25 (20)
Diabetes mellitus—no. (%)	—	116 (91)
Diabetes mellitus type 1—no. (%)	0 (0.0)	—
Diabetes mellitus type 2—no. (%)	115 (99)	—
Latent autoimmune diabetes of the adult—no. (%)	1 (1)	—
No diabetes—no. (%)	0 (0.0)	—
HbA1c (%)—*m* ± SD	8.5 ± 1.4	40 (31)
HbA1c (mmol/mol)—*m* ± SD	79 ± 16	40 (31)
Medication use	—	—
Metformin—no. (%)	85 (98)	87 (68)
Sulfonylurea (SU)—no. (%)	22 (100)	22 (17)
Other oral glucose‐lowering agent(s)—no. (%)	47 (81)	58 (45)
Insulin therapy—no. (%)	33 (65)	51 (40)
Insulin dosage (international units, IU)—*m* ± SD	60 ± 48	14 (42)
SGLT2 inhibitor used—no. (%)	—	128 (100)
Empagliflozin	52 (41)	—
Canagliflozin	40 (31)	—
Dapagliflozin	27 (21)	—
Ertugliflozin	1 (1)	—
Undefined	8 (6)	—
Last dosage of SGLT2 inhibitor–no. (%)	—	77 (60)
3 days before surgery	4 (5)	—
2 days before surgery	12 (16)	—
1 day before surgery	36 (47)	—
Taken on the day of surgery	10 (13)	—
Not ceased	15 (19)	—
Type of surgery–no. (%)		127 (99)
Bariatric	27 (21)	—
Cardiac	23 (18)	—
Abdominal	17 (13)	—
Orthopaedic	10 (8)	—
Neurological	10 (8)	—
Other	40 (32)	—

*Note:* Baseline characteristics.

Abbreviations: *m* = mean; no. = number; SD = standard deviation.

### 3.4 SGLT2i Management

**FIGURE 2:**
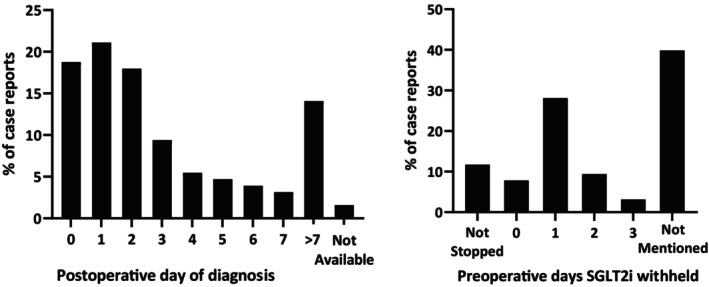
Number of preoperative days SGLT2i withheld and number of postoperative days until SAPKA occurred. Left: Number of days SGLT2i were withheld before surgery. Right: Number of days after surgery, the occurrence of SAPKA was detected.

The most commonly prescribed SGLT2i were empagliflozin (*n* = 52; 41%), canagliflozin (*n* = 40; 31%) and dapagliflozin (*n* = 27; 21%) (Table 2). Whether the SGLT2i were withheld preoperatively was not mentioned in 51 (40%) cases; in the other cases, the SGLT2i were continued in 25 (20%), stopped 1 day before surgery in 36 (28%), and stopped 2 days or more before surgery in 16 (13%) cases (Figure 2, left).

### 3.5 SAPKA Presentation

The duration between surgery and presentation of the potential SAPKA cases was available in 126 (98.4%) cases, with a median of 2 days (range: 0–120 days) after surgery (Figure 2, right; Table 4). All patients presented with acidosis (pH < 7.3), with a mean pH of 7.15 ± 0.1; full biochemical parameters, including bicarbonate, base excess, anion gap, lactate and ketone levels, are summarised in Table 3. Bicarbonate was reported in 103 (80.5%) of cases and was ≤ 22 mmol/L in 102 of these, with one case reporting a value above this threshold. These findings are reported descriptively to illustrate the variability in biochemical documentation across published case reports; no cases were excluded on the basis of these parameters. The base excess was reported in 31 (24%) cases, all of which were < −5 mmol/L. In 106 cases (83%), acidosis could be classified as metabolic based on either bicarbonate or base excess. pCO_2_ was reported in 38 cases (30%), with a pCO_2_ of 3.26 ± 1.6 kPa. The anion gap was reported or calculated in 88 cases (69%), with a mean of 23.2 ± 6.6 mEq/L; of these, 80 cases (91%) exceeded the 16 mEq/L threshold. In the 95 cases (74%) where sufficient electrolyte data were available to classify acid–base status, a high anion gap was present in 93 (98%). Lactate was reported in 67 cases (52% of the total cohort), with a mean of 1.48 ± 1.1 mmol/L, and was elevated (> 2 mmol/L) in 10 of these cases. Where elevated, lactate was considered by the expert panel as a potential indicator of alternative causes of metabolic acidosis, such as sepsis or haemodynamic compromise, and contributed accordingly to the likelihood classification. Elevated ketones were measured in blood in 106 cases (83%), in urine in 61 cases (49%) or in both in 38 cases (30%) (Table 3). Of those with serum ketone measurements, levels exceeded 3.0 mmol/L in 87% of cases, with a mean serum ketone level of 6.9 ± 6.6 mmol/L. Urinary ketones were present in all 61 cases where tested (100% of those tested; 48% of total cohort), with a mean urinary ketone concentration of 15.6 ± 7.4 mmol/L in the subset where quantitative values were reported. The mean serum glucose concentration measured at diagnosis was 9.89 ± 2.5 mmol/L (178.02 ± 45 mg/dL). A summary of clinical symptoms is provided in Supporting Information S4. The most frequent symptoms included tachycardia (*n* = 28, 21.9%), nausea (*n* = 24, 18.8%), emesis (*n* = 23, 18.0%) and tachypnoea (*n* = 22, 17.2%). In 42 cases (33%), no clinical symptoms were reported.

**TABLE 3:** Laboratory results of patients with SAPKA.

Laboratory results	Result	Data available—no. (%)
pH—*m* ± SD	7.15 ± 0.1	128 (100)
pCO_2_ (kPa)—*m* ± SD	3.26 ± 1.6	38 (30)
Bicarbonate (mmol/L)—*m* ± SD	10.41 ± 5.1	103 (80)
Na^+^ (mmol/L)—*m* ± SD	139.2 ± 7.2	35 (27)
K^+^ (mmol/L)—*m* ± SD	4.1 ± 0.7	43 (34)
Cl^−^ (mmol/L)—*m* ± SD	104.8 ± 21.9	25 (19)
High anion gap—*n* (%)	93 (98)	95 (74)
Anion gap (mEq/L)—*m* ± SD	23.2 ± 6.6	88 (69)
Base excess—*m* ± SD	−16.69 ± 6.0	31 (24)
Glucose serum (mmol/L)—*m* ± SD	9.89 ± 2.5	85 (66)
Lactate (mmol/L)—*m* ± SD	1.48 ± 1.1	67 (52)
Serum ketones elevated—*n* (%)	94 (87)	106 (83)
Serum ketones (mmol/L)—*m* ± SD	6.68 ± 6.6	95 (74)
Urinary ketones present—*n* (%)	61 (100)	61 (48)
Urine ketones stick value—*m* ± SD	2.56 ± 1.1	31 (24)
Urinary ketones (mg/dL)—*m* ± SD	15.6 ± 7.4	13 (10)
Urinary glucose (mmol/L)—*m* ± SD	176.2 ± 48.7	85 (66)

*Note:* Laboratory values. All percentages in the Result column reflect the proportion of cases with available data; total cohort proportions are reported in the Results text. High anion gap was classified based on available electrolyte data.

Abbreviations: *m* = mean; *n* = number; SD = standard deviation.

### 3.6 SAPKA Diagnosis

The expert panel could not validate the diagnosis of SAPKA in 10 (8%) of the 128 cases based on insufficient data to support the diagnosis and the time between the last SGLT2i use and the onset of ketoacidosis. The remaining 118 cases were classified as likely, possible, and unlikely SAPKA in 53 (41%), 38 (30%) and 27 (21%), respectively. We observed a ‘substantial level’ of agreement between the assessors (*k* = 0.38, 95% CI = 0.23–0.54, *p* < 0.001) and a third assessor was involved for 32 cases of disagreement between the first two reviewers. The most frequent reasons for classifying cases as unlikely SAPKA included: timing between ketoacidosis and SGLT2i use (*n* = 53), severely diminished postoperative intake (*n* = 30) and preoperative intake (*n* = 23), reduced or withheld insulin treatment (*n* = 10) and presence of an infection (*n* = 8) (Supporting Information S5).

### 3.7 Treatment and Outcomes

Insulin was administered in 104 of 105 cases with available data (99%; 81% of the total cohort), dextrose or glucose infusion in 58 of 66 cases with available data (88%; 45% of total cohort), and bicarbonate infusion in 28 of 44 cases with available data (64%; 22% of total cohort) (Table 4). The resolution time of ketoacidosis was reported for 77 cases (60%), with a mean of 88 ± 84 h (range: 4–504 h), reflecting considerable variability in reported recovery trajectories. Thirteen patients (10%) resumed their SGLT2i after discharge from the hospital, with most cases not reporting whether the SGLT2i were discontinued indefinitely. In cases where insulin was not administered, resolution was achieved with supportive care, including fluid resuscitation and SGLT2i discontinuation, consistent with a milder metabolic disturbance in these instances. Two fatal outcomes (1.5%) were reported. One involved a patient who suffered from a cerebral infarction occurring shortly after brain surgery, and another involved a person who suffered from a myocardial infarction after elective hip surgery [18, 19]. According to our expert panel, these two fatal outcomes were deemed unrelated to SAPKA.

**TABLE 4:** Treatment details for patients with SAPKA.

Treatment details	Total	Data available—no. (%)
Days after surgery diagnosis was made—median, IQR	2, 1–5	126 (98)
Day of surgery—no. (%)	24 (19)	—
Day after surgery—no. (%)	27 (21)	—
2 Days after surgery—no. (%)	23 (18)	—
3 Days after surgery—no. (%)	12 (10)	—
Within 1 week—no. (%)	108 (86)	—
More than 1 week—no. (%)	18 (14)	—
Fluid resuscitation—no. (%)	71 (100)	71 (55)
Insulin—no. (%)	104 (99)	105 (82)
Glucose/Dextrose—no. (%)	58 (88)	66 (52)
Bicarbonate—no. (%)	28 (64)	44 (34)
Hours to resolve—*m* ± SD	88.2 ± 83.5	77 (60)
Fatal outcome—no. (%)	2 (2)	128 (100)
Restarted SGLT2 inhibitor after normalization—no. (%)	13 (28)	47 (37)

*Note:* Treatment details found within the case reports.

Abbreviations: IQR = interquartile range; no. = number; SD = standard deviation.

## 4 Discussion

This systematic review aimed to critically evaluate the role of SGLT2i in SAPKA case reports. Of the 128 included cases, SAPKA was considered likely or possible in 71% and unlikely in 29%, based on expert panel adjudication. A key finding of this review is therefore twofold: a subset of well‐documented cases was validated by our expert panel, confirming that SAPKA is a clinically relevant perioperative complication. Meanwhile, the substantial proportion of poorly documented or implausibly attributed cases demonstrates that SAPKA is also over‐reported in the published literature. The high proportion of unlikely classifications was in part attributable to the variable quality of reporting: 32% of cases were scored as poor quality, with frequent omission of essential details on the timing of SGLT2i use, surgery, and ketoacidosis onset. Reported outcomes after SAPKA were associated with significant morbidity, with ketosis and metabolic acidosis resolving following recognition and initiation of insulin treatment, though ICU admission was required in a substantial proportion of reported cases.

The confirmation that SGLT2i were likely involved in the significant metabolic derangements reported emphasises the importance for clinicians to be aware of this complication, especially in light of the rising prevalence of this treatment in the perioperative population [20]. To prevent this complication, the FDA and international scientific societies advise preoperatively withholding all SGLT2i for 3–4 days [21, 22, 23, 24]. In contrast, a British multidisciplinary consensus statement recently advised omitting SGLT2i on the day before and the day of a procedure only [24]. Despite these recommendations, SGLT2i cannot be withheld in case of emergency surgery. Recently, an extensive retrospective analysis of such cases (*n* = 2607) found no increased risk of postoperative ketoacidosis [25]. This led the authors to conclude that relaxation of perioperative SGLT2i management could be considered.

The expert panel considered a third of the cases unlikely to be related to the treatment with an SGLT2i. This was partly due to the insufficient quality of reporting, with the omission of essential details on the timing of treatment, surgery and onset of symptoms, or related to confounding factors required to differentiate other causes of (metabolic) acidosis. This highlights the importance of paying attention to detail in reporting cases of rare or novel complications. Notably, temporal mismatch between surgery and ketoacidosis onset was the most common reason for an ‘unlikely SAPKA’ classification by the expert panel, cited in 53 cases. This highlights a recurring pattern of over‐attribution in the published literature, where authors reported cases as perioperative SAPKA despite intervals between surgery and ketoacidosis onset that render a causal relationship with surgical stress implausible. The absence of a predefined postoperative time window for inclusion was intentional, as it allowed this discrepancy to be made visible and quantified. Complicating the assessment of SGLT2i involvement in cases of ketoacidosis is the observation that increases in ketonemia have been observed in cardiac surgery patients not receiving SGLT2i treatment [14]. The surgical stress response, including catecholamine release, increases insulin resistance, promoting a shift to ketogenesis in the usually fasted patient [26]. As a result, ketonemia has been observed in patients with and without T2D in the absence of SGLT2i use [14]. Combined with other precipitating factors such as infection, insufficient insulin treatment or strongly impaired nutritional intake, ketoacidosis may occur irrespective of SGLT2i use.

We cannot exclude the possibility of bias in the sample of reported cases. Still, from this evaluation of currently published cases, SAPKA was associated with considerable morbidity, including prolonged hospital stays and, in previous case series, ICU admission rates of 40%–60% [10, 11]. Reporting bias in case reports may underrepresent severe outcomes; nevertheless, with prompt recognition, cases resolved with insulin treatment and supportive care. In most cases, patients received insulin treatment to suppress ketogenesis, according to current clinical practice for DKA, with normalisation of metabolic acidosis. Perioperative insulin and glucose infusion has been suggested as a preventive strategy for SAPKA and as a potential alternative to preoperative SGLT2i cessation [27, 28]. This should be interpreted in the context that ketosis is common in the perioperative setting regardless of SGLT2i use, as previously demonstrated in non‐SGLT2i‐treated cardiac surgery patients [14]. Whether perioperative insulin and glucose infusion could serve as a preventive strategy—and potentially reduce the need for routine preoperative SGLT2i cessation—is a hypothesis that warrants prospective evaluation but cannot be addressed by the present case report data. It is notable that SAPKA occurred in a subset of cases where SGLT2i had been discontinued 2 or more days before surgery, suggesting that guideline‐recommended cessation periods do not universally prevent the complication [28]. This may reflect individual pharmacokinetic variability, residual effects on ketogenesis beyond the standard washout period or the contribution of additional perioperative precipitating factors.

The clinical relevance of this review extends beyond a descriptive catalogue of reported cases. Perioperative practice is evolving, with growing evidence that continuation of SGLT2i may be preferable to cessation in certain patient groups—particularly those prescribed SGLT2i for heart failure, in whom discontinuation carries its own short‐term cardiovascular risks, including rebound deterioration in cardiac function [28]. An important complicating factor in assessing the true SAPKA risk is that surgery itself promotes ketogenesis through the stress response, independent of SGLT2i use or diabetes status. Moderate perioperative ketonaemia should therefore not be automatically attributed to SGLT2i treatment; multiple coexisting factors—including surgical stress, fasting, infection and insulin management—may each contribute and must be evaluated in aggregate. This complexity underpins our use of a structured expert panel rather than a simple algorithmic classification: the case‐by‐case weighing of multiple perioperative factors requires clinical expert judgement that a fixed algorithm cannot adequately replicate.

### 4.1 Strengths and Limitations

This systematic review of case reports benefits significantly from the input of our six endocrinology experts, who possess in‐depth knowledge of metabolic derangements, which helps place SAPKA into perspective at a clinician level and is highly relevant in light of the rising numbers of SGLT2i use due to its benefits for the treatment of chronic kidney disease and heart failure. As previously done by Stades et al. [29], expert panel evaluations of metformin‐induced lactic acidosis illustrate how a patient's clinical characteristics are essential in determining the underlying causes of a clinical presentation and that alternative explanations and contributing factors must not be ignored.

Limitations are inherent in the collection and analysis of case reports. Due to the increased awareness in recent years, it is challenging to infer the actual prevalence or incidence of SAPKA, while reporting bias limits the conclusions on the severity of outcomes from this complication. The annual number of published case reports has declined since its peak in 2021, with only five cases reported in 2023 and two in 2024. This most likely reflects a reduction in the perceived novelty of SAPKA as a reportable finding rather than a true decrease in incidence, which cannot be determined from case report data alone. Furthermore, we observed a significant lack of quality in many case reports, which restricts the validity of classifying metabolic derangements as SAPKA. When classifying cases as ‘unlikely SAPKA’, the expert panel distinguished between routine perioperative fasting—which is an expected component of surgical care and a recognised trigger for SAPKA in SGLT2i‐treated patients—and prolonged fasting of 24 h or more arising from causes independent of the surgical procedure (such as severe preoperative malnutrition, protracted postoperative ileus or excessive dietary restriction). The latter represents an established independent cause of starvation ketoacidosis and, when present as the predominant explanatory factor, was used to support an ‘unlikely SAPKA’ classification. Routine surgical fasting was not considered sufficient grounds to exclude SGLT2i as a contributing cause. Furthermore, readers should note that the expert panel questionnaire was structured to assess whether ketoacidosis was related to the surgical insult itself (Q3). This means the panel was primarily evaluating surgery‐triggered SAPKA, rather than the full spectrum of unavoidable perioperative factors—including fasting and infection—that may contribute to SAPKA in SGLT2i‐treated patients. The stated aims, which describe evaluation of ‘all factors related to SAPKA’, are therefore broader than what the questionnaire structure captures, and findings should be interpreted with this limitation in mind.

## 5 Conclusion

In the 128 case reports assessed, SAPKA was considered likely or possibly due to SGLT2i treatment in 71% (91/128) of cases. However, SAPKA is challenging to diagnose due to many confounding factors in the perioperative period. Nonetheless, treatment is effective, and reported use of SGLT2i in the perioperative period warrants awareness for SAPKA among perioperative clinicians. These findings confirm SAPKA as a genuine perioperative complication warranting clinical awareness and provide descriptive, hypothesis‐generating insights to guide future prospective research. Whether early recognition and treatment could safely reduce or replace the need for routine preoperative SGLT2i cessation cannot be determined from case report data and requires prospective evaluation.

## Author Contributions

L.I.P. Snel: protocol design search strategy, design, formal analysis, screening, writing of the original draft. X. Li: protocol design, screening reviewer. F. Jamaludin: literature search. A.H. Hulst: reviewer, screening. J. Hermanides: reviewer. B. Preckel: reviewer. D.H. van Raalte: reviewer, expert panel member. S.E. Siegelaar: Expert panel member. F. Holleman: expert panel member. T.M. Vriesendorp: expert panel member. J.H. DeVries: expert panel member. J.B.L. Hoekstra: expert panel member.

## Funding

This project has received funding from the European Union's Horizon 2020 research and innovation program under the Marie Skłodowska‐Curie grant agreement No. 101024833.

## Conflicts of Interest

D.H.v.R. has served as a consultant and received honoraria from Boehringer Ingelheim and Lilly, Merck, Sanofi and AstraZeneca, and has received research operating funds from Boehringer Ingelheim and Lilly Diabetes Alliance and AstraZeneca; all honoraria are paid to his employer (Amsterdam University Medical Centres, Amsterdam). The other authors declare no conflicts of interest.

## Supporting information


**Supporting Information: S1.** Search Strategy.
**Supporting Information: S2** Questionnaire answer options.
**Supporting Information: S3** Included articles.
**Supporting Information: S4** Observed symptoms.
**Supporting Information: S5** Reviewer responses.

## Data Availability

The data that support the findings of this study are available from the corresponding author upon reasonable request.
