# Supplementary material for: A Systematic Review and Expert Evaluation of Perioperative SGLT2 Inhibitor‐Associated Ketoacidosis Case Reports
Source: Acta Anaesthesiol Scand. 2026 May 13;70:e70254. doi: 10.1111/aas.70254 (PMC13172662; doi:10.1111/aas.70254)
Supplement: Supplementary file 1 — Supporting Information: S1. Search Strategy. Supporting Information: S2 Questionnaire answer options. Supporting Information: S3 Included articles. Supporting Information: S4 Observed symptoms. Supporting Information: S5 Reviewer responses. [file AAS-70-0-s001.docx]

**Supplements**

**Supplement 1: Search Strategy performed on 18-06-2024**

Medline: 186 results

1. acidosis/ or ketosis/ or diabetic ketoacidosis/ or hydroxybutyrates/ or 3-hydroxybutyric acid/ or exp ketones/ or exp ketone bodies/
2. ketoacidos* or ketosis or ketonemia* or ketonaemia* or acetonuria* or ketoacidemia* or ketoacidaemia* or acetonemia* or acetonaemia* or ketone* or 3-hydroxybutyrate* or hydroxybutyrate* or hydroxybutyric acid* or acidos*). ti,ab,kf.
3. 1 or 2
4. exp Sodium-Glucose Transporter 2 Inhibitors/
5. SGLT2* or SGLT-2* or sodium-glucose transporter 2* or sodium-glucose-cotransporter-2* or sodium-glucose-co-transporter-2* or sodium-glucose transporters 2* or sodium-glucose-cotransporters-2* or sodium-glucose-co-transporters-2* or dapagliflozin or empagliflozin or canagliflozin or ertugliflozin).ti,ab,kf.
6. 4 or 5
7. exp Surgical Procedures, Operative/ or exp perioperative care/ or exp perioperative period/
8. (surger* or surgical* or surgeon* or operat* or perioperat* or peri-operat* or preoperat* or pre-operat* or intraoperat* or intra-operat* or postoperat* or post-operat*).ti,ab,kf
9. 7 or 8
10. 3 and 6 and 9

Embase: 1662 results

1. acidosis/ or ketosis/ or diabetic ketoacidosis/ or hydroxybutyrates/ or 3-hydroxybutyric acid/ or exp ketones/ or exp ketone bodies/
2. ketoacidos* or ketosis or ketonemia* or ketonaemia* or acetonuria* or ketoacidemia* or ketoacidaemia* or acetonemia* or acetonaemia* or ketone* or 3-hydroxybutyrate* or hydroxybutyrate* or hydroxybutyric acid* or acidos*).ti,ab,kf.
3. 1 or 2
4. exp Sodium-Glucose Transporter 2 Inhibitors/
5. SGLT2* or SGLT-2* or sodium-glucose transporter 2* or sodium-glucose-cotransporter-2* or sodium-glucose-co-transporter-2* or sodium-glucose transporters 2* or sodium-glucose-cotransporters-2* or sodium-glucose-co-transporters-2* or dapagliflozin or empagliflozin or canagliflozin or ertugliflozin).ti,ab,kf.
6. 4 or 5
7. exp Surgical Procedures, Operative/ or exp perioperative care/ or exp perioperative period/
8. (surger* or surgical* or surgeon* or operat* or perioperat* or peri-operat* or preoperat* or pre-operat* or intraoperat* or intra-operat* or postoperat* or post-operat*).ti,ab,kf
9. 7 or 8
10. 3 and 6 and 9

3. CINAHL (Ebsco): 38 results

( (MH "Acidosis+") OR (MH "3-Hydroxybutyric Acid+") OR (MH "Ketones") ) OR ( TI ( ketoacidos* or ketosis or ketonemia* or ketonaemia* or acetonuria* or ketoacidemia* or ketoacidaemia* or acetonemia* or acetonaemia* or ketone* or 3-hydroxybutyrate* or hydroxybutyrate* or hydroxybutyric acid* or acidos* ) OR AB ( ketoacidos* or ketosis or ketonemia* or ketonaemia* or acetonuria* or ketoacidemia* or ketoacidaemia* or acetonemia* or acetonaemia* or ketone* or 3-hydroxybutyrate* or hydroxybutyrate* or hydroxybutyric acid* or acidos* ) )

AND

(MH "Sodium-Glucose Co-Transporter 2 Inhibitors+") OR TI ( SGLT2* or SGLT-2* or sodium-glucose transporter 2* or sodium-glucose-cotransporter-2* or sodium-glucose-co-transporter-2* or sodium-glucose transporters 2* or sodium-glucose-cotransporters-2* or sodium-glucose-co-transporters-2* or dapagliflozin or empagliflozin or canagliflozin or ertugliflozin ) OR AB ( SGLT2* or SGLT-2* or sodium-glucose transporter 2* or sodium-glucose-cotransporter-2* or sodium-glucose-co-transporter-2* or sodium-glucose transporters 2* or sodium-glucose-cotransporters-2* or sodium-glucose-co-transporters-2* or dapagliflozin or empagliflozin or canagliflozin or ertugliflozin )

AND

(MH "Surgery, Operative+") OR (MH "Perioperative Care+") OR ( (MH "Intraoperative Care+") OR (MH "Intraoperative Period") ) OR TI ( surger* or surgical* or surgeon* or operat* or perioperat* or peri-operat* or preoperat* or pre-operat* or intraoperat* or intra-operat* or postoperat* or post-operat* ) OR AB ( surger* or surgical* or surgeon* or operat* or perioperat* or peri-operat* or preoperat* or pre-operat* or intraoperat* or intra-operat* or postoperat* or post-operat* )

Cochrane 16 results

#1 (ketoacidos* or ketosis or ketonemia* or ketonaemia* or acetonuria* or ketoacidemia* or ketoacidaemia* or acetonemia* or acetonaemia* or ketone* or hydroxybutyrate* or hydroxybutyric* or acidos*):ti,ab,kw

#2 (SGLT2* or SGLT-2* or sodium-glucose transporter 2* or sodium-glucose-cotransporter-2* or sodium-glucose-co-transporter-2* or sodium-glucose transporters 2* or sodium-glucose-cotransporters-2* or sodium-glucose-co-transporters-2* or dapagliflozin or empagliflozin or canagliflozin or ertugliflozin):ti,ab,kw

#3 (surger* or surgical* or surgeon* or operat* or perioperat* or peri-operat* or preoperat* or pre-operat* or intraoperat* or intra-operat* or postoperat* or post-operat*):ti,ab,kw

#4 #1 and #2 and #3

**Supplement 2: Questionnaire answer lists: for Q2, Q3,**

List of options available for questionnaire Q2

Insufficient data to support the diagnosis

Does not fulfil criteria for a ketoacidaemia

Other causes of ketosis are more likely

Another cause of high anion gap metabolic acidosis is more likely

The respiratory component more likely contributed to the acidosis

SGLT-2i and last dosage and time of onset ketoacidosis do not match

List of options available for questionnaire Q3

Abdominal/Gastric surgery

Alcohol use disorder

Anti-depressant medication use

Anti-psychotic medications use

Corticosteroid use

Gastro-intestinal Motility problem

Hepatic disease

Hepatic or Pancreatic surgery/Disease

Impaired kidney function (Acute/Chronic)

Infection

Insulin discontinuation/dose reduction

Missed diagnosis of DM1 or LADA

Myocardial infarction

Non-compliance in medication use

Persisting emesis/vomiting

Postoperative very low-calorie intake or fasting

Preoperative very low-calorie intake or fasting

Stroke or Transient ischemic attack(TIA)

Trauma

Use of non-SGLT-2 inhibitor oral glucose-lowering medication(s)

Vasopressor use

The time of surgery and occurrence of SAPKA do not match

**Supplement 3: Included articles (n=93)**

1. Banakh I, Kung R, Gupta S, Matthiesson K, Tiruvoipati R. Euglycemic diabetic ketoacidosis in association with dapagliflozin use after gastric sleeve surgery in a patient with type II diabetes mellitus. Clinical case reports. 2019;7(5):1087-90.

2. Bobart SA, Gleason B, Martinez N, Norris K, Williams SF. Euglycemic ketoacidosis caused by sodium-glucose cotransporter 2 inhibitors: A case report. Annals of Internal Medicine. 2016;165(7):530-2.

3. Bonanni FB, Fei P, Fitzpatrick LL. Normoglycemic ketoacidosis in a postoperative gastric bypass patient taking canagliflozin. Surgery for obesity and related diseases : official journal of the American Society for Bariatric Surgery. 2016;12(1):e11-e2.

4. Buck S, Russo SG. [Perioperative Ketoacidosis under SGLT2-Inhibitors]. Perioperative Ketoazidose unter SGLT2-Hemmer. 2021;56(10):704-8.

5. Darwish AM. Metabolic Acidosis in Postsurgical Patient on Canagliflozin and Metformin: A Case Report. A&A practice. 2019;12(7):221-2.

6. Fisker F, Moller N, Lauritzen ES. [Sodium-glucose-cotransporter 2 inhibitor-associated euglycaemic diabetic ketoacidosis]. Ugeskrift for laeger. 2019;181(21).

7. Gonzalez ME, Velez JCQ. A case of perioperative euglycemic ketoacidosis and concomitant non-anion gap metabolic acidosis and AKI associated with canagliflozin. Journal of the American Society of Nephrology. 2017;28:937-8.

8. Goto S, Ishikawa J-Y, Idei M, Iwabuchi M, Namekawa M, Nomura T. Life-Threatening Complications Related to Delayed Diagnosis of Euglycemic Diabetic Ketoacidosis Associated with Sodium-Glucose Cotransporter-2 Inhibitors: A Report of 2 Cases. The American journal of case reports. 2021;22:e929773.

9. Howard J, Wiegley N, Madan N. Severe euglycemic diabetic ketoacidosis secondary to sodium-glucose cotransporter 2 (SGLT2) inhibitor: A diagnostic challenge. Journal of the American Society of Nephrology. 2019;30:1198.

10. Karakaya Z, Topal FE, Topal F, Payza U, Akyol PY. Euglisemic diabetic ketoacidotic coma caused by dapagliflozin. American Journal of Emergency Medicine. 2018;36(11):2136.e1-.e2.

11. Kohli J, Goldfarb S. Quiz: Metabolic Acidosis in a Patient With Type 2 Diabetes. American Journal of Kidney Diseases. 2017;69(6):A11-A3.

12. Yeoh HL, Lee M, Pan WJ, Ong HY. Case of sodium-glucose cotransporter-2 inhibitor-associated euglycaemic diabetic ketoacidosis. BMJ Case Reports. 2021;14(8):e235953.

13. Wood T, Pang AJ, Hallet J, Greig P. Euglycaemic ketoacidosis in a postoperative Whipple patient using canaglifozin. BMJ case reports. 2016;2016:216607.

14. Wang KM, Isom RT. EUGLYCEMIC KETOACIDOSIS IN A POSTOPERATIVE PATIENT TAKING EMPAGLIFLOZIN. American Journal of Kidney Diseases. 2019;73(5):745.

15. Smith A, Holtrop J, Sadoun M. Postoperative Euglycemic Diabetic Ketoacidosis in a Patient With SGLT-2 Inhibitor Use and Recent Sleeve Gastrectomy. Cureus. 2021;13(4):e14297.

16. Siddiqi A, Bollineni M. Euglycemic diabetic ketoacidosis associated with canagliflozin use. Endocrine Practice. 2020;26(SUPPL 2):111.

17. Sampani E, Sarafidis P, Dimitriadis C, Kasimatis E, Daikidou D, Bantis K, et al. Severe euglycemic diabetic ketoacidosis of multifactorial etiology in a type 2 diabetic patient treated with empagliflozin: case report and literature review. BMC nephrology. 2020;21(1):276.

18. Pontes JPJ, de Melo CS, Arantes FBB, de Souza Ramos JTG, Modolo NSP, Navarro E Lima LH. Perioperative euglycemic diabetic ketoacidosis following use of SGLT-2 inhibitors after cardiac surgery. Journal of clinical anesthesia. 2021;71:110201.

19. Pang J, Malik RQ, Ho SY, Saha S, Hussain N. Sodium Glucose Co-Transporter 2 (SGLT2) Inhibitor: How well do we know the side effect profile in ITU? Should Current perioperative diabetes guideline be challenged? Journal of the Intensive Care Society. 2018;19(2 Supplement 1):122.

20. Milne L, Bryant C. Severe euglycaemic ketoacidosis with SGLT2 inhibitor use in the perioperative period. Anaesthesia. 2019;74(Supplement 4):12.

21. Menhem M, Kunstadt R. Euglycemic diabetic ketoacidosis in type 2 diabetes mellitus following sleeve gastrectomy. Diabetes. 2017;66(Supplement 1):A347.

22. Lau A, Bruce S, Wang E, Ree R, Rondi K, Chau A. Perioperative implications of sodium-glucose cotransporter-2 inhibitors: a case series of euglycemic diabetic ketoacidosis in three patients after cardiac surgery. Implications perioperatoires des inhibiteurs du cotransporteur sodium-glucose de type 2 : une serie de cas d'acidocetose diabetique euglycemique chez trois patients suite a une chirurgie cardiaque. 2018;65(2):188-93.

23. Kartha AP, Irimpan J, Thomas DE, Kumar L. Euglycaemic ketoacidosis after abdominal surgery: Implications of SGLT2 inhibitors for anaesthesiologists - A case study. Indian J Anaesth. 2023;67(9):840-1.

24. Fleyshman R. Canagliflozin (Invokana) Induced Ketoacidosis and Proximal Renal Tubular Acidosis in the Setting of Euglycemia following Sugery. Journal of the American Society of Nephrology. 2016;Conference: Kidney Week 2016.

25. Rathi SN, Patil KN, Swami SS, Konnur SL. Euglycaemic diabetic ketoacidosis after bariatric surgery: A Near-Miss!!! Indian J Anaesth. 2022;66(11):812-3.

26. Andalib A, Elbahrawy A, Alshlwi S, Alkhamis A, Hu W, Demyttenaere S, et al. Diabetic ketoacidosis following bariatric surgery in patients with type 2 diabetes. Diabetes Care. 2016;39(8):e121-e2.

27. Candelario N, Wykretowicz J. The DKA that wasn't: A case of euglycemic diabetic ketoacidosis due to empagliflozin. Oxford Medical Case Reports. 2016;2016(7):144-6.

28. Chacko B, Whitley M, Beckmann U, Murray K, Rowley M. Postoperative euglycaemic diabetic ketoacidosis associated with sodium-glucose cotransporter-2 inhibitors (gliflozins): a report of two cases and review of the literature. Anaesthesia and intensive care. 2018;46(2):215-9.

29. Di Palma R, Vanderpuye A, Satara J, Ceronie B, Trenfield S. A case of empagliflozin-induced euglycaemic diabetic ketoacidosis post-off pump coronary artery bypass surgery. Diabetic Medicine. 2020;37(SUPPL 1):78.

30. Dizon S, Keely EJ, Malcolm J, Arnaout A. Insights Into the Recognition and Management of SGLT2-Inhibitor-Associated Ketoacidosis: It's Not Just Euglycemic Diabetic Ketoacidosis. Canadian Journal of Diabetes. 2017;41(5):499-503.

31. Hamill CF, Hunter SJ. A case of Euglycaemic DKA in a perioperative patient taking an SGLT2 inhibitor for type 2 Diabetes. Irish Journal of Medical Science. 2017;186(9 Supplement 1):S367-S8.

32. Hawkins AM, Jackson, R.V., White, H., Vardesh, D.L. SGLT2-Inhibitor induced euglycemic ketoacidosis in acute surgical patients. Edorium journal. 2017.

33. Hogenkamp CGJB, Veneman SK, Backus EMJM, Veneman TF. Euglycaemic diabetic ketoacidosis as a severe complication of dapagliflozin. Netherlands Journal of Critical Care. 2022;30(2):71-3.

34. Horikoshi R, Kitamura T, Miyaji K. Ketoacidosis related to sodium glucose cotransporter 2 inhibitors after emergency coronary surgery. Interactive cardiovascular and thoracic surgery. 2019:323-4.

35. Hung K-C, Chen J-Y, Chu C-C, Sun C-K. Perioperative Surgical Pleth Index as a Stress Indicator for the Prevention of Perioperative Euglycemic Diabetic Ketoacidosis. , <Blank>: Springer Nature; 2021. p. 673-4.

36. Iqbal QZ, Mishiyev D, Niazi MR, Zia Z, Sattar SBA, Jahanghir A, Quyyumi S. SGLT-2 Inhibitors-a Culprit of Diabetic Ketoacidosis Postbariatric Surgery. Case reports in critical care. 2020;2020:8817829.

37. Kapila V, Topf J. Sodium-Glucose Co-transporter 2 Inhibitor-Associated Euglycemic Diabetic Ketoacidosis After Bariatric Surgery: A Case and Literature Review. Cureus. 2021;13(8):e17093.

38. Yared KE, Mancini GJ. Euglycemic Diabetic Ketoacidosis Associated With Use of SGLT2 Inhibitor After Laparoscopic Roux-en-Y Gastric Bypass. The American surgeon. 2021;87(12):1997-9.

39. Yap SD, Hamblin PS, Bach L, Ekinci E, Wong R. Sodium glucose co-transporter-2 inhibitor-induced diabetic ketoacidosis following tooth extraction: improving awareness among dental practitioners. Australian dental journal. 2021;66(4):444-7.

40. Wohlrab P, Kainz M, Schiferer A, Zapletal B, Tschernko E. Euglycemic Diabetic Ketoacidosis After Cardiac Surgery in a Patient Treated With Empagliflozin for Type 2 Diabetes Mellitus. Journal of cardiothoracic and vascular anesthesia. 2022;36(7):2066-9.

41. Alabdaljabar M. A, Khaled M. Euglycemic Diabetic Ketoacidosis in a Sedated Patient after Coronary Artery Bypass Grafting: A Case Report and Literature Review. 2021:1-6.

42. Ahn T, Teloken P, Burke P. Sodium-glucose linked transporter 2 inhibitor associated postoperative euglycaemic diabetic ketoacidosis: an important consideration for all surgeons. ANZ journal of surgery. 2019;89(7-8):982.

43. Aggarwal A, Jain A, Sachdeva S, Kulairi ZI. Prolonged Glucosuria With Sodium-Glucose Cotransporter-2 (SGLT2) Inhibitors: A Case Report and Review of Literature. Cureus. 2020;12(11):e11554.

44. Amianda EA, Gavigan TS, Talishinskiy T, Ewing DR, Schmidt HJ. Two cases of euglycemic diabetic ketoacidosis after bariatric surgery associated with sodium-glucose cotransporter-2 inhibitor use. Obesity surgery. 2021;31(8):3848-50.

45. Bolimunta P. Ns. Case report. Endocrine Reviews. 2018;Endocrine Society's 100th Annual Meeting and Expo: 214-5.

46. Brown F, McColl T. Euglycemic Diabetic Ketoacidosis Secondary to Dapagliflozin Use: A Case Report. The Journal of emergency medicine. 2018;54(1):109-11.

47. Bteich F, Daher G, Kapoor A, Charbek E, Kamel G. Post-surgical Euglycemic Diabetic Ketoacidosis in a Patient on Empagliflozin in the Intensive Care Unit. Cureus. 2019;11(4):e4496.

48. Chong ZM, Stirling A, Sandeep T. Sodium-glucose cotransporter 2 (SGLT-2): Cause of prolonged euglycaemic ketoacidosis and ketonaemia following general anaesthesia? Diabetic Medicine. 2018;35(Supplement 1):100.

49. Curanaj FM, Seley J. Euglycemic DKA in a patient with type 2 diabetes on an SGLT2 inhibitor: Is it a zebra? Endocrine Practice. 2020;26(SUPPL 2):142.

50. Evans LC, Salahuddin S. Just sweet enough? A case of euglycaemic ketoacidosis 'incidentally' found in a post-surgical patient. Diabetic Medicine. 2018;35(Supplement 1):105.

51. Gallardo HAG, Gomez CAG, Mendoza AJC, Antezana J, Hernandez NG, Estrada MC. EUGLYCEMIC DIABETIC KETOACIDOSIS AFTER TRANSCATHETER AORTIC VALVE REPLACEMENT. A BITTERSWEET DIAGNOSIS. Journal of the American College of Cardiology. 2022;79(9 Supplement):2937.

52. Gomez-Sanchez CM, Wu BX, Gotts JE, Chang RW. Euglycemic diabetic ketoacidosis following major vascular surgery is a new item on the differential for postoperative acidosis. Journal of vascular surgery cases and innovative techniques. 2021;7(4):778-80.

53. Hoffman C, Green M, Megafu O. Sodium-glucose linked transporter 2 inhibitor-associated perioperative euglycaemic diabetic ketoacidosis: a case for a perioperative guideline. Anaesthesia and intensive care. 2017;45(6):758.

54. Isaacs M, Tonks KT, Greenfield JR. Euglycaemic diabetic ketoacidosis in patients using sodium-glucose co-transporter 2 inhibitors. Internal Medicine Journal. 2017;47(6):701-4.

55. Ito T, Sugasawa G, Suzuki F, Sunada M, Iwamuro K, Nakano T, et al. Insulin and glucose infusion could prevent euglycemic diabetic ketoacidosis associated with sodium-glucose cotransporter 2 inhibitors. Indian journal of thoracic and cardiovascular surgery. 2022;38(1):87-91.

56. Kitahara C, Morita S, Kishimoto S, Matsuno S, Uraki S, Takeshima K, et al. Early detection of euglycemic ketoacidosis during thoracic surgery associated with empagliflozin in a patient with type 2 diabetes: A case report. Journal of diabetes investigation. 2021;12(4):664-7.

57. Kuchay MS, Mishra SK, Mehta Y. Empagliflozin induced euglycemic diabetic ketoacidosis in a patient undergoing coronary artery bypass graft despite discontinuation of the drug 48 hours before the surgery. Diabetes & metabolic syndrome. 2021;15(3):909-11.

58. Kumar A, Routray SK, Arora S. 160 MYSTERY OF UNRESPONSIVE SEVERE METABOLIC ACIDOSIS. American Journal of Kidney Diseases. 2021;77(4):617.

59. Wong YC, Liu K-L, Lee C-L. Postoperative extremity gangrene in a patient with type 2 diabetes taking SGLT2 inhibitors: A case report. Medicine. 2021;100(16):e25590.

60. Wang R, Kave B, McIlroy E, Kyi M, Colman PG, Fourlanos S. Metabolic outcomes in patients with diabetes mellitus administered SGLT2 inhibitors immediately before emergency or elective surgery: single centre experience and recommendations. British journal of anaesthesia. 2021;127(1):e5-e7.

61. Wang CT, Chang CW, Lu YC, Lam HC, Ku SJ, Tseng KB, Kao YH. An atypical hyperosmolar hyperglycemic state and diabetic ketoacidosis induced by sodium-glucose cotransporter-2 inhibitors: A case report. Journal of the Formosan Medical Association. 2020;119(8):1325-8.

62. Van Niekerk CJ, Wallace JD, Takata MC, Yu RC. Euglycemic diabetic ketoacidosis in bariatric surgery patients taking canagliflozin. Surgical Endoscopy and Other Interventional Techniques. 2017;31(Supplement 1):S180.

63. Vadi S, Lad V, Kapoor D. Perioperative Implication of Sodium-glucose Cotransporter-2 Inhibitor in a Patient Following Major Surgery. Indian journal of critical care medicine : peer-reviewed, official publication of Indian Society of Critical Care Medicine. 2021;25(8):958-9.

64. Tsai MK, Chen GL, Chuang TJ. Dapagliflozin-induced postoperative euglycemic diabetic ketoacidosis: Two cases. Journal of Internal Medicine of Taiwan. 2019;30(1):47-50.

65. Tansey DJ, Pazderska A, Healy ML. Please don't sugarcoat it: An avoidable case of Euglycaemic DKA in the setting of a SGLT-2 Inhibitor. Irish Journal of Medical Science. 2019;188(Supplement 9):S248-S9.

66. Smyth CC, Collins M, McCarthy J, Galvin S. The catabolic triad: case report of fasting, major cardiac surgery and sodium-glucose co-transporter 2 inhibitors leading to perioperative euglycaemic ketoacidosis. Interactive cardiovascular and thoracic surgery. 2021;33(3):494-5.

67. Seger CD, Xing H, Wang L, Shin JS. Intraoperative Diagnosis of Sodium-Glucose Cotransporter 2 Inhibitor-Associated Euglycemic Diabetic Ketoacidosis: A Case Report. A&A practice. 2021;15(1):e01380.

68. Schwartz S, Vahdatpour C, Chaudhari R. POST SURGICAL BYPASS OF DIABETIC KETOACIDOSIS PROTOCOL. Chest. 2019;156(4 Supplement):A799.

69. Schempf J, Chow LS, Harindhanavudhi T. Uncovering Autoimmune Diabetes in a Patient With Euglycemic Diabetic Ketoacidosis. American Journal of the Medical Sciences. 2020;360(3):307-8.

70. Saha S. Euglycemic DKA-an evil of SGLT2 inhibitors, a newer age OHA. Journal of the Intensive Care Society. 2020;21(2 SUPPL):72.

71. Rizo IM, Apovian CM. Diabetic ketoacidosis post bariatric surgery. Frontiers in Endocrinology. 2019;10(JAN).

72. Rafey MF, Butt A, Coffey B, Reddington L, Devitt A, Lappin D, Finucane FM. Prolonged acidosis is a feature of SGLT2i-induced euglycaemic diabetic ketoacidosis. Endocrinology, diabetes & metabolism case reports. 2019;2019:19-0087.

73. Theodore D, Pongracz B, Titus B. Euglycemic ketoacidosis in a postoperative deformity correction spine surgery patient: a case report. Journal of Emergency and Critical Care Medicine. 2018;2:83-.

74. Osafehinti DA, Okoli OJ, Karam JG. A Case of SGLT2 Inhibitor-Associated Euglycemic Diabetic Ketoacidosis Following Coronary Artery Bypass Surgery. AACE clinical case reports. 2021;7(1):20-2.

75. Noubleau AMA, Narang DK. Development of euglycemic diabetic ketoacidosis in postbariatric surgery patients with type 2 diabetes mellitus on canaglifozin. Endocrine Reviews. 2018;39(2 Supplement 1).

76. Mossler J, Hwang S. A saccharin case of diabetic ketoacidosis. Journal of Hospital Medicine. 2018;13(4 Supplement 1).

77. Mehta PB, Robinson A, Burkhardt D, Rushakoff RJ. Inpatient Perioperative Euglycemic Diabetic Ketoacidosis Due to Sodium-Glucose Cotransporter-2 Inhibitors - Lessons From a Case Series and Strategies to Decrease Incidence. Endocrine practice : official journal of the American College of Endocrinology and the American Association of Clinical Endocrinologists. 2022.

78. Mackintosh C, Tewari A, Siegel J, Wang RD, Freeman W. Postoperative Euglycemic Diabetic Ketoacidosis and Encephalopathy Related to SGLT-2 Inhibitors: A Case Report and Discussion of Diabetes Treatment and "Sweet Pee Encephalopathy" in Perioperative Hospital Management. The Neurohospitalist. 2020;10(1):51-4.

79. Luo X, Ji R, Lu W, Zhu H, Li L, Hu J. Dapagliflozin-Associated Euglycemic Diabetic Ketoacidosis in a Patient Who Underwent Surgery for Pancreatic Carcinoma: A Case Report. Frontiers in surgery. 2022;9:769041.

80. Lindsay PJ, Gibson LE, Bittner EA, Berg S, Chang MG. Sodium-glucose cotransporter-2 (SGLT2) inhibitor-induced euglycemic diabetic ketoacidosis complicating the perioperative management of a patient with type 2 diabetes mellitus (T2DM) and Fournier's gangrene: A case report. International journal of surgery case reports. 2020;77:463-6.

81. Li J, Dizon S, Arnaout A. SGLT-2 inhibitors & ketacidosis in bariatric patients: A cases series. Endocrine Reviews. 2016;37(2 Supplement 1).

82. Lane S, Paskar D, Hamed S, Goffi A. When Guidelines Fail: Euglycemic Diabetic Ketoacidosis After Bariatric Surgery in a Patient Taking a Sodium-Glucose Cotransporter-2 Inhibitor: A Case Report. A&A practice. 2018;11(2):46-8.

83. Nishida A, Ogawa O, Takizawa H. Detection of Euglycemic Diabetic Ketoacidosis During Thoracic Surgery 75 Hours After Empagliflozin Discontinuation. Cureus. 2022;14(10):e29974.

84. Ritchie DT, Dixon J. SGLT-2 inhibitor associated euglycaemic diabetic ketoacidosis in an orthopaedic trauma patient. BMJ Case Rep. 2022;15(9).

85. Chaudhry A, Roels C, Lee J. Sodium-Glucose Cotransporter-2 Inhibitor-associated Euglycemic Diabetic Ketoacidosis: Lessons From a Case Series of 4 Patients Undergoing Coronary Artery Bypass Grafting Surgery. Can J Diabetes. 2022;46(8):843-50.

86. Choi CH, Singh S, Cheung AT, Vanneman M, Madhok J. Prolonged Postoperative Euglycemic Diabetic Ketoacidosis in a Lung Transplant Recipient With Preoperative SGLT2 Inhibitor Use. J Cardiothorac Vasc Anesth. 2024;38(7):1539-42.

87. Figaro RC, A., Shah, V., ; Cordeiro, K.,; Song, N.; Shenkman, D; Cely, C; Vera-Arroyo, A. A POST-SURGICAL CASE OF EUGLYCEMIC DIABETIC KETOACIDOSIS INDUCED BY EMPAGLIFLOZIN. Critical Care Medicine. 2023;51.

88. Hammond DB, Ingram CC. Euglycemic diabetic ketoacidosis in a patient on sodium-glucose cotransporter 2 inhibitors. JAAPA. 2023;36(9):1-3.

89. Nallu R, ; Azmath, M.,; , Aftab, H.,: y Kantorovich, V. A Case of Euglycemic DKA With SGLT 2 Inhibitor Use A Diagnostic and Therapeutic Dilemma. Journal Endocrine Society. 2021;5.

90. Sitina M, Lukes M, Sramek V. Empagliflozin-associated postoperative mixed metabolic acidosis. Case report and review of pathogenesis. BMC Endocr Disord. 2023;23(1):81.

91. J. T. A Case of Euglycemic Diabetic Ketoacidosis After

Distal Pancreatectomy. Journal Endocrine Society. 2023;6(Supplement_1).

92. M S. SIGNIFICANTLY DELAYED ONSET OF EUGLYCEMIC DIABETES KETOACIDOSIS WITH SGLT2I THERAPY: A CASE REPOrt. Critical Care Medicine. 2023.

93. Wang Q, Wu K, Luo X, Dong X, Liu W, Tang Z, Zhang B. Dapagliflozin-Associated Euglycemic Diabetic Ketoacidosis Presenting With Severe Abdominal Pain Mimicking Acute Peritonitis. Cureus. 2022;14(2):e22229.

**Supplement 4: Observed symptoms described at the time of diagnosis:**

| Symptoms | Frequency |
| --- | --- |
| Airway symptoms |  |
| Dyspnoea | 9 |
| Shortness of breath | 9 |
| Low oxygen saturation | 2 |
| Tachypnoea | 22 |
| Pulmonary oedema | 1 |
| Kussmaul's breathing pattern | 3 |
| Circulatory symptoms |  |
| Tachycardia | 28 |
| Shock | 1 |
| Hypotension | 6 |
| Palpitations | 1 |
| Atrial fibrillation | 1 |
| Cardiac arrhythmia | 3 |
| Gastrointestinal symptoms |  |
| Emesis | 23 |
| Constipation | 1 |
| Abdominal distention | 4 |
| Abdominal pain | 14 |
| Nausea | 24 |
| Ileus like symptoms | 2 |
| Dysphagia | 1 |
| Dyspepsia | 2 |
| Diarrhoea | 1 |
| Decreased appetite/intake | 8 |
| Neurocognitive symptoms |  |
| Dizziness | 2 |
| Delirium/confusion | 9 |
| reduced vigilance/responsiveness | 6 |
| Pre-syncope | 1 |
| Encephalopathy | 1 |
| Headache | 1 |
| Increased agitation or irritable | 3 |
| Aphasia | 2 |
| Lethargy/weakness | 16 |
| Urological symptoms |  |
| Increased urine output/Polyuria | 7 |
| Pollakiuria |  |
| Acute Kidney Injury | 1 |
| Polydipsia/dehydration | 9 |
| Others |  |
| Backpain | 2 |
| Fever | 4 |
| Fruity odour on breath | 1 |
| Chills | 1 |
| General malaise | 7 |
| No symptoms described | 41 |

**Supplement 5a: Number of times an answer was selected by any reviewer.**

| Q2 Reason why it is not euglycemic ketoacidosis | N=54 |
| --- | --- |
| Insufficient data to support the diagnosis | 21 |
| The last dosage of the SGLT-2 inhibitor and the time of onset of ketoacidosis do not match. | 19 |
| Another cause for high anion gap metabolic acidosis is more likely. | 8 |
| Does not meet the criteria for ketoacidemia | 6 |

Table S5a: Reasons why the case report was found not likely to be a ketoacidosis with involvement of SGLT2i.

**Supplement 5b: Number of times an answer was selected by any reviewer.**

| Q4 Reasons given ketoacidosis was not associated with surgery.  (maximum 3 options per case per expert) | N=158 |
| --- | --- |
| The time of surgery and occurrence of DKA does not match | 53 |
| Postoperative low-calorie intake or fasting | 30 |
| Preoperative low-calorie intake or fasting | 23 |
| Insulin discontinuation/dose reduction | 10 |
| Infection | 8 |
| Impaired kidney function (Acute/Chronic) | 7 |
| Hepatic or Pancreatic surgery/Disease | 5 |
| Persisting emesis/vomiting | 5 |
| Vasopressor use | 4 |
| Gastro-intestinal Motility problem | 3 |
| Stroke or Transient ischemic attack(TIA) | 2 |
| Use of non-SGLT-2i's oral glucose-lowering medication(s) | 2 |
| Abdominal/gastric surgery | 2 |
| Corticosteroid use | 1 |
| Missed diagnosis of DM1 or LADA | 1 |
| Myocardial infarction | 1 |
| Non-compliance in medication use | 1 |

Table S5b. Reasons why the case report was found not to be directly associated with the performed surgery.
